# Burden of cardiovascular disease in Brazil, 1996–2023: a retrospective descriptive study of the epidemiology and impact on public healthcare

**DOI:** 10.1016/j.lana.2026.101674

**Published:** 2026-10-14

**Authors:** Mellyely Carolina Basílio-Soares, João Sérgio Fonseca Guimarães, Ana Luíza Matos de Oliveira, André Gustavo Oliveira, Thiago Verano-Braga

**Affiliations:** a Departamento de Fisiologia e Biofísica, Instituto de Ciências Biológicas, Universidade Federal de Minas Gerais, 31270-901, Belo Horizonte, Minas Gerais, Brazil; b United Nations, Resident Coordinator Office in Guatemala, Guatemala City, Guatemala

**Keywords:** Cardiovascular diseases, Acute myocardial infarction, Public health, Burden of disease Brazil

## Abstract

**Background:**

Cardiovascular diseases (CVD) are the leading cause of death worldwide, and their epidemiology is correlated with genetic predisposition and exposure to risk. Brazil is a developing country with a vast territory, which leads to structural inequalities. We aimed to comprehensively characterise the epidemiology and burden of CVD in Brazil between 1996 and 2023, examining temporal trends, regional disparities, demographic patterns, healthcare costs, and heart transplantation.

**Methods:**

We conducted a nationwide retrospective descriptive ecological study using aggregated administrative data from the Brazilian Unified Health System (SUS), Brazil's publicly funded national health system. Mortality, hospitalization, and heart transplantation data from 1996 to 2023 were analysed according to sex, race/ethnicity, age, and geographic region. We estimated mortality rates for the major CVD, hospital length of stay, healthcare expenditures, and heart transplantation activity.

**Findings:**

Acute myocardial infarction remained the leading cause of cardiovascular mortality throughout the study period, while hypertensive diseases overtook heart failure after 2016 as the second leading cause of death. The overall CVD mortality rate—calculated as the number of CVD-related deaths divided by the total population and expressed as a percentage—increased from 7.3% in 1998 to 8.3% in 2023, peaking at 10.1% in 2021. Marked regional, age-, sex-, and race/ethnicity-related disparities were observed, including the complete absence of heart transplantation in the North region throughout the study period. Although hospitalization rates remained relatively stable, CVD-related healthcare expenditures and heart transplantation activity increased over time.

**Interpretation:**

CVD remains a major and growing public health challenge in Brazil, with substantial regional and demographic inequalities in disease burden and access to specialised care. These findings highlight the need for targeted public health strategies and more equitable allocation of healthcare resources to reduce cardiovascular morbidity and mortality.

**Funding:**

This study was funded by CNPq, CAPES, FAPEMIG, INCT.

**Research in context**

**Evidence before this study**

Data recently published by the World Health Organization show that cardiovascular diseases (CVD) are the leading cause of death worldwide, with acute myocardial infarction and stroke among the most prevalent conditions. In light of this, we conducted a literature review between January and April 2025, examining epidemiological data on CVD published during the previous five years (2021–2025) using Google Scholar and PubMed. Search terms included “myocardial infarction,” “heart transplantation,” “hypertensive diseases,” and “hospital costs associated with cardiovascular diseases.” Articles published in Portuguese and English were included, while publications in other languages were excluded. The available evidence was fragmented across studies, and key aspects of the burden of CVD, including its temporal evolution and mortality patterns, were not consistently characterised. Although CVD is well established as a leading cause of mortality, information available across health systems and registries remains fragmented and can be difficult to integrate and interpret.

**Added value of this study**

This study extends existing evidence by providing an integrated assessment of the epidemiological and health-system burden of CVD in Brazil. By considering mortality, healthcare utilisation, economic burden, access to specialised care, and demographic and regional inequalities within a single framework, it brings together dimensions that have often been examined separately in previous studies. This approach complements existing modelled estimates of CVD burden with evidence derived directly from nationwide administrative health records and provides a broader understanding of how the burden of cardiovascular disease and access to care vary across Brazil. These findings help address important gaps in the existing evidence and provide a basis for healthcare planning, resource allocation, and future research aimed at reducing cardiovascular health inequalities.

**Implications of all the available evidence**

CVD remains a major cause of mortality and healthcare burden in Brazil. Taken together with existing evidence, our findings highlight substantial demographic and regional inequalities in the burden of CVD and access to cardiovascular care in Brazil. These inequalities underscore the importance of public health strategies that account for the distinct needs of different populations and regions. The available evidence supports strengthening cardiovascular prevention and surveillance, improving equitable access to specialised care, and guiding region-specific healthcare planning and resource allocation. Future research should investigate the factors underlying these disparities and evaluate strategies to reduce inequalities in cardiovascular outcomes and access to care.

## Introduction

According to data from the World Health Organization (WHO), cardiovascular diseases (CVD) remain the leading cause of global mortality, accounting for approximately 19.8 million deaths in 2022. CVD arise from a complex interplay of genetic, environmental, and socioeconomic determinants, with modifiable risk factors such as obesity, unhealthy dietary habits, psychosocial stress, physical inactivity, and tobacco use playing a major role in their development and progression.^1^, ^2^, ^3^

In a global analysis, data from the Global Health Data Exchange (GHDx), used to estimate the Global Burden of Disease (GBD) study, indicate that CVD-related mortality increased by approximately 18.5% between 1990 and 2023. In Latin America and the Caribbean, the corresponding increase was 8.33%, whereas Brazil experienced an even greater rise of 11.53%. These findings underscore the growing burden of CVD over recent decades, likely driven by population aging, demographic and epidemiological transitions, lifestyle changes, and persistent socioeconomic and healthcare disparities.

Within the International Classification of Diseases (ICD), the ICD-10 version places CVD in Chapter IX. This coding system standardizes clinical and epidemiological records, ensuring greater consistency in data collection and analysis. Identifying the population groups most affected is crucial for shaping public health policies and for designing effective strategies aimed at prevention and the promotion of cardiovascular health.

Among CVD, ischemic heart disease (I25), hypertensive diseases (I10–I15), chronic rheumatic heart disease (I05–I09), acute myocardial infarction (I21), cardiomyopathies (I42), and heart failure (I50) stand out, as they represent some of the most prevalent conditions and are among the leading causes of morbidity and mortality worldwide.^4^, ^5^, ^6^ The high prevalence of these CVD imposes a substantial economic burden on healthcare systems, increasing both public and private expenditures through greater demand for hospitalizations, diagnostic procedures, chronic pharmacological therapy, and, in advanced cases, high-complexity interventions such as heart transplantation.^7^

In Brazil, health information is managed through the Department of Informatics of the Unified Health System (DATASUS), the official information system of the Brazilian Unified Health System (SUS), established in 1991 to integrate and disseminate national health data. Despite its comprehensive database, DATASUS still presents limitations in data visualization and the integration of multiple variables, hindering more comprehensive epidemiological analyses. In this study, we transformed these large-scale datasets into epidemiological and graphical representations spanning 1996–2023, enabling the investigation of regional, ethnic, sex-, and age-related patterns of cardiovascular disease mortality and morbidity, as well as their economic burden on the Brazilian public healthcare system. Complementing previous GBD analyses in Brazil,^8^^,^^9^ which provide modelled estimates of disease burden, our study directly analyses official nationwide administrative health databases to integrate mortality, hospitalizations, healthcare expenditures, heart transplantation, and demographic and regional disparities within a single nationwide assessment. This integrated health system perspective is not provided by previous nationwide studies and enables a more comprehensive evaluation of healthcare utilization, resource allocation, and inequalities in cardiovascular care across Brazil.

## Methods

### Study design and data collection

This nationwide retrospective ecological time-series study analysed the burden of cardiovascular diseases (CVD) in Brazil between 1996 and 2023. The study population comprised all registered deaths due to diseases of the circulatory system in Brazil and all hospitalisations for these conditions recorded in the Brazilian Unified Health System (SUS) during the study period. Analyses were based exclusively on aggregated administrative data, including mortality (number of deaths and mortality rates), morbidity (hospital admissions, length of stay, and healthcare expenditures), and heart transplantation. The most prevalent disorders primarily involving the heart and cardiovascular system were subsequently analysed according to geographic region, sex, age, and race/ethnicity.

Aggregated data were available either at the national level or stratified by population subgroup (region, sex, age, and race/ethnicity). Mortality data were obtained from the Mortality Information System (SIM), hospitalisation data from the Hospital Information System (SIH/SUS), and heart transplantation data from the National Transplant System (SNT), all managed by the Brazilian Ministry of Health. Diseases were identified according to Chapter IX (Diseases of the Circulatory System) of the International Classification of Diseases, 10th Revision (ICD-10). The following diseases were included in this study: I00 Rheumatic fever without mention of cardiac involvement, I01 Rheumatic fever with cardiac involvement, I02 Rheumatic chorea, I05 Rheumatic mitral valve diseases, I06 Rheumatic diseases of the aortic valve, I07 Rheumatic diseases of the tricuspid valve, I08 Multiple valve diseases, I09 Other rheumatic diseases of the heart, I10 Essential (primary) hypertension, I11 Hypertensive heart disease, I12 Hypertensive renal disease, I13 Hypertensive heart and kidney disease, I15 Secondary hypertension, I20 Angina pectoris, I21 Acute myocardial infarction, I22 Recurrent myocardial infarction, I24 Other acute ischemic heart disease, I25 Chronic ischemic heart disease, I26 Pulmonary embolism, I27 Other forms of pulmonary heart disease, I28 Other diseases of the pulmonary vessels, I30 Acute pericarditis, I31 Other pericardial diseases, I33 Acute and subacute endocarditis, I34 Non-rheumatic mitral valve disorders, I35 Non-rheumatic aortic valve disorders, I36 Non-rheumatic disorders of the tricuspid valve, I37 Disorders of the pulmonary valve, I38 Unspecified valve endocarditis, I40 Acute myocarditis, I42 Cardiomyopathies, I44 Atrioventricular and left bundle branch block, I45 Other conduction disorders, I46 Cardiac arrest, I47 Paroxysmal tachycardia, I48 Atrial flutter and fibrillation, I49 Other cardiac arrhythmias, I50 Heart failure, I51 Complications of heart disease and poorly defined heart conditions, I60 Subarachnoid hemorrhage, I61 Intracerebral hemorrhage, I62 Other non-traumatic intracranial hemorrhages, I63 Cerebral infarction, I64 Stroke, not specified as hemorrhagic or ischemic, I66 Occlusion and stenosis of cerebral arteries not resulting in cerebral infarction, I67 Other cerebrovascular diseases, I69 Sequelae of cerebrovascular diseases, I70 Atherosclerosis, I71 Aortic aneurysm and dissection, I72 Other aneurysms, I73 Other peripheral vascular diseases, I74 Arterial embolism and thrombosis, I77 Other diseases of arteries and arterioles, and I78 Diseases of the capillaries. The analysed variables (Table S1) and parameters (Table S2) can be visualised in the Supplemental Material.

Mortality data are consolidated by the Department of Health Situation Analysis of the Health Surveillance Secretariat in collaboration with the State and Municipal Health Departments. Death certificates completed by the certifying physician are collected from local civil registry offices and subsequently entered into the Mortality Information System (SIM). Therefore, mortality data are not derived directly from hospital electronic health records but from standardized death certificates, following the rules established by the World Health Organization for certification and coding of causes of death. Because death registration is mandatory in Brazil, the SIM provides nationwide coverage of mortality data. Mortality data were analysed from 1996 to 2023, corresponding to the period during which diseases were classified according to the ICD-10.

Morbidity data were obtained from the Hospital Information System of the Brazilian Unified Health System (SIH/SUS). Hospital admission records are submitted by public hospitals and private hospitals contracted by the SUS to municipal and state health authorities and are subsequently consolidated by DATASUS. Consequently, the database includes hospitalizations financed by the SUS, which provides healthcare to approximately 70% of the Brazilian population. Previous studies have shown that SUS coverage is broadly representative across demographic and geographic subgroups, with 64% and 61% of all female and male hospitalizations, respectively, occurring in SUS-participating hospitals. Regional coverage also remains substantial, ranging from 55.6% in the Southeast to 77.1% in the Northeast.^10^ Admission data were identified using ICD-10 Chapter IX (Diseases of the Circulatory System) codes (Table S3, Supplemental Material), and analyses included hospital admissions, admission rates, length of stay, and healthcare expenditures from 1998 to 2023. The database contains aggregated administrative records rather than individual-level data, precluding patient follow-up, differentiation between readmissions and incident cases, and assessment of comorbidities. Furthermore, hospital records are not uniformly generated from electronic health records before submission to the SIH/SUS, as data collection and reporting procedures vary across institutions.

The regional divisions used in this study follow the official classification established by the Brazilian Institute of Geography and Statistics (IBGE), based on geographical, demographic, and economic criteria. Brazil is divided into five regions: North, Northeast, Midwest, Southeast, and South. Mortality, hospital admissions, heart transplantation, and intercurrence data are presented throughout the manuscript as rates per 100,000 population and are referred to in the figures as “Deaths,” “Admissions,” “Heart transplants,” and “Intercurrences,” respectively. Rates were calculated using population counts from the national censuses and intercensal population estimates provided by the IBGE. Age standardisation was performed using the WHO World Standard Population (2000–2025).^11^

Sociodemographic data for Brazilian states, including population counts from the most recent census and gross domestic product (GDP), were obtained from the Brazilian Institute of Geography and Statistics (IBGE).^12^^,^^13^ GDP per capita was calculated by dividing each state's GDP by its population. Municipal Human Development Index (MHDI) data were obtained from the United Nations Development Programme (UNDP).^14^

Cost values were adjusted for inflation using the Extended National Consumer Price Index (*Índice Nacional de Preços ao Consumidor Amplo*, IPCA) calculated by IBGE. All costs are reported in Brazilian reais (BRL) adjusted to 2026 values. For international comparison, values were converted from BRL to US dollars (USD) using the nominal exchange rate provided by the Brazilian Central Bank on 28 July 2026 (1 BRL = 0.1954003 USD). The conversion was performed solely to facilitate international interpretation of the reported costs and was not adjusted for purchasing power parity (PPP). Monetary values reported in the text and figures were rounded for simplicity.

### Analysis

Data analysis was carried out through R (version 4.4.2) and RStudio (2024.04.2 Build 764). Descriptive analyses were performed for all CVD included in ICD-10 Chapter IX and listed above. For disease-specific analyses, we focused on the cardiovascular conditions responsible for the greatest mortality burden during the study period, namely acute myocardial infarction, hypertensive diseases, hypertensive heart disease, heart failure, chronic rheumatic heart disease, chronic ischaemic heart disease, and cardiomyopathies. To identify significant temporal changes in nationwide CVD mortality trends, we performed Joinpoint regression using the R package “segmented”.^15^ The outcome variable was the annual age-standardized mortality rate for all diseases included in ICD-10 Chapter IX (Diseases of the Circulatory System), aggregated at the national level for each year between 1996 and 2023 (Table S3, Supplemental Material). The model used calendar year as the independent variable and fitted piecewise linear segments to the mortality rate over time. Davies' test was used to identify statistically significant changes in slope (joinpoints) during the study period.

To calculate age-specific and age-standardized CVD mortality rates, the entire study population (men and women combined) was stratified into age groups according to the Brazilian Ministry of Health classification^16^: 0–4, 5–9, 10–14, 15–19, 20–29, 30–39, 40–49, 50–59, 60–69, 70–79, and ≥80 years. Bayesian time-series models of age-specific mortality rates were fitted using the R package surveil^17^ through Hamiltonian Markov chain Monte Carlo (MCMC) sampling, and results are reported with 95% confidence intervals (CIs). Age-specific mortality rates were then weighted according to the WHO World Standard Population (2000–2025) to obtain age-standardized mortality rates. Sex-specific analyses were performed separately and are presented independently from the age-standardized analyses.

Because only aggregated administrative data were available, information on individual socioeconomic characteristics and pre-existing medical conditions was unavailable, precluding adjustment for these potential confounding factors. Descriptive mortality rates and other summary measures were calculated directly from nationwide administrative records representing the target population rather than a sample and, therefore, are presented without hypothesis testing or CIs based on sampling variability. Statistical inference was restricted to model-based analyses, for which uncertainty arises from parameter estimation.

### Ethics statement

This study was based exclusively on publicly available, aggregated, and de-identified administrative data obtained from DATASUS. No individual-level or identifiable patient information was accessed.

### Role of funding source

The funding sources were not involved in the study design, data acquisition, analysis or interpretation of the data, or preparation of the manuscript.

## Results

### Mortality and morbidity from cardiovascular diseases in Brazil by region, type of heart disease, sex, race and ethnicity, and age

We evaluated the evolution of cardiovascular diseases (CVD) in Brazil between 1996 and 2023 using DATASUS data. Registered deaths peaked in 2022 at approximately 195 per 100,000 inhabitants (Fig. 1A), likely reflecting the impact of the COVID-19 pandemic, which was associated with underreporting, worsening CVD outcomes, increased sedentary behaviour, poorer dietary habits, reduced healthcare utilization, and interruptions to ongoing treatments.^18^ In contrast, age-standardized mortality rates declined between 2000 and 2023 (Fig. 1A, inset).

**Fig. 1:**
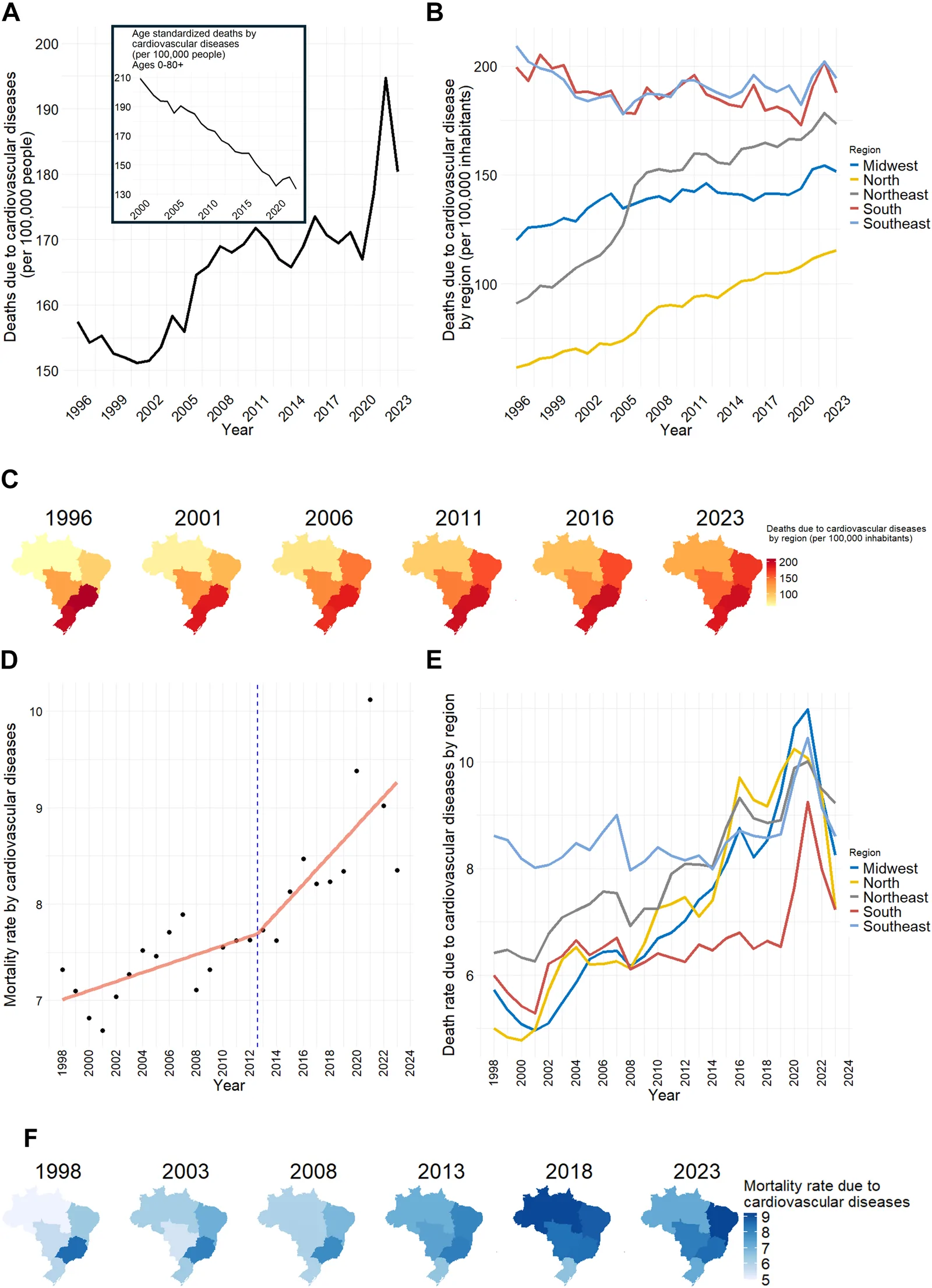
**Cardiovascular disease (CVD) mortality in Brazil from 1996 to 2023.** (**A**) Absolute number of CVD deaths and crude mortality rate (per 100,000 inhabitants) obtained annually from DATASUS. Inset: age-standardised mortality rate from 2000 to 2023. (**B**) Annual number of CVD deaths according to the Brazilian five geographic regions Midwest, Northeast, North, Southeast, and South. (**C**) Geographic distribution of CVD deaths across Brazil, with colour intensity representing the number of deaths per 100,000 inhabitants. (**D**) Joinpoint regression analysis of the CVD mortality rate from 1998 to 2023, showing the observed values and fitted temporal trends. (**E**) Temporal evolution of CVD mortality rates according to Brazil's geographic regions. (**F**) Geographic distribution of CVD mortality rates across Brazil at selected time points, with colour intensity representing the mortality rate.

To investigate regional heterogeneity in the burden of CVD, we compared mortality trends across the five Brazilian regions (South, Southeast, Midwest, Northeast and North), which differ substantially in their demographic and socioeconomic characteristics (Table S4, Supplemental Material). From the 2000s onward, the number of CVD-related deaths increased steadily in the Midwest, North, and Northeast regions, whereas the Southeast and South regions maintained consistently high but relatively stable numbers of deaths (Fig. 1B and C). The overall mortality rate also increased over the study period, rising from 7.3% in 1998 to 8.3% in 2023, with a steeper upward trend after 2012 and a peak of approximately 10.1% in 2021 (Fig. 1D). Marked changes were also observed in the regional distribution of CVD mortality rates over time. In 2008, the Southeast region exhibited the highest mortality rate, whereas by 2022 the Midwest ranked first, with the North and Northeast reaching similarly elevated levels. By 2023, the Northeast had become the region with the highest CVD mortality rate, while the South consistently exhibited the lowest rates from 2008 onward (Fig. 1E and F).

We next examined the cardiovascular conditions responsible for the greatest mortality burden during the study period (Fig. 2A and B). These disease-specific analyses were restricted to disorders primarily involving the heart and cardiovascular system. Consequently, cerebrovascular diseases (e.g., I64 stroke, I61 nontraumatic intracerebral haemorrhage, I67 other cerebrovascular diseases, and I69 sequelae of cerebrovascular disease), as well as other conditions included in ICD-10 Chapter IX (e.g., I01 rheumatic fever with heart involvement), were not included, despite their clinical importance.

**Fig. 2:**
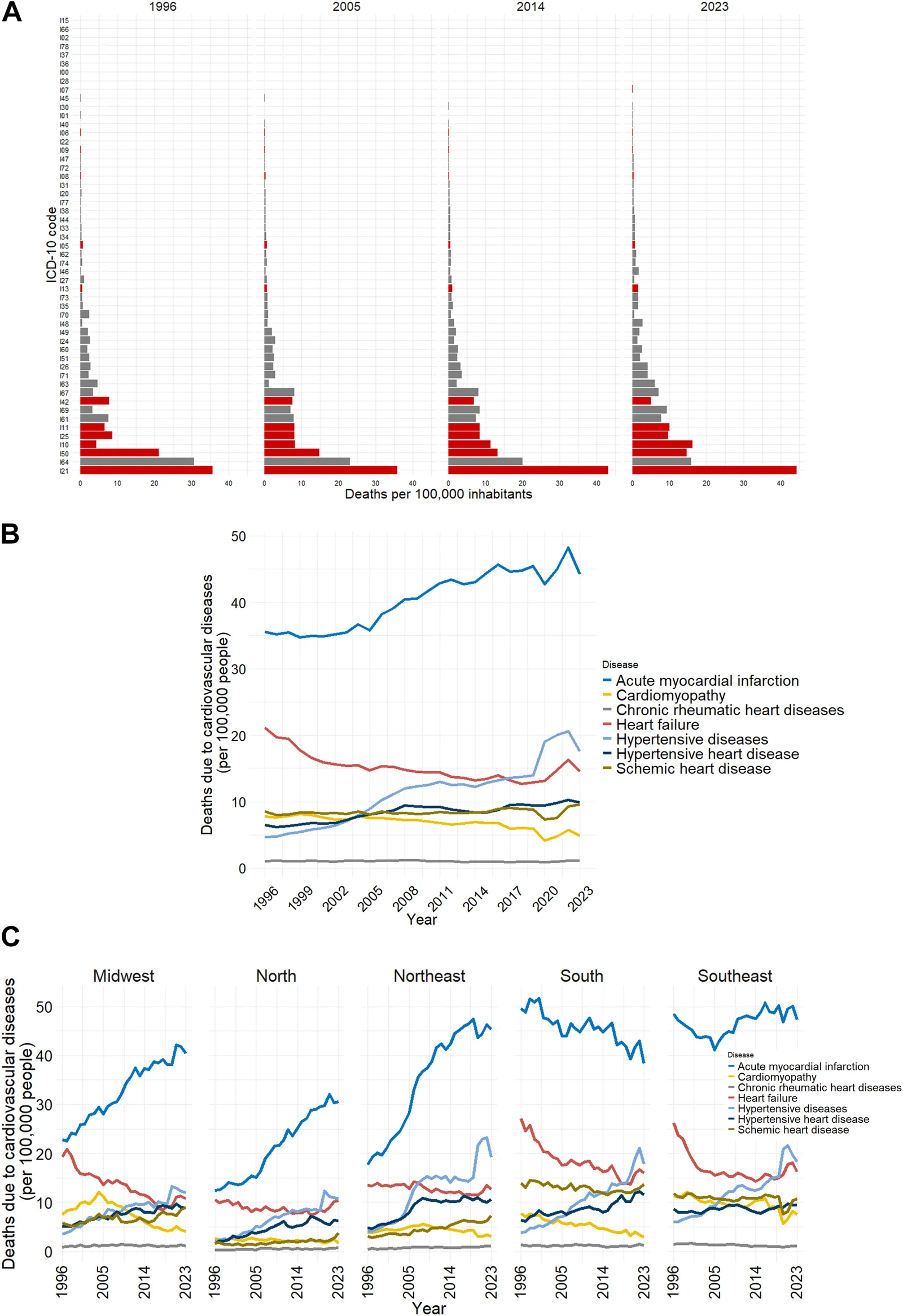
**Temporal evolution of the leading causes of cardiovascular disease (CVD) mortality in Brazil (1996**–**2023).** (**A**) Data was collected from the DATASUS platform and the classification was made according to the 10th revision of the International Classification of Diseases (ICD-10), Chapter IX Diseases of the circulatory system. Each code indicates a specific disease, as follows: I15 Secondary hypertension, I02 Rheumatic chorea, I66 Occlusion and stenosis of cerebral arteries not resulting in cerebral infarction, I78 Diseases of the capillaries, I37 Disorders of the pulmonary valve, I36 Non-rheumatic disorders of the tricuspid valve, I00 Rheumatic fever without mention of cardiac involvement, I28 Other diseases of the pulmonary vessels, I07 Rheumatic diseases of the tricuspid valve, I45 Other conduction disorders, I30 Acute pericarditis, I01 Rheumatic fever with cardiac involvement, I40 Acute myocarditis, I06 Rheumatic diseases of the aortic valve, I22 Recurrent myocardial infarction, I09 Other rheumatic diseases of the heart, I47 Paroxysmal tachycardia, I72 Other Aneurysms, I08 Multiple valve diseases, I31 Other pericardial diseases, I20 Angina pectoris, I77 Other diseases of arteries and arterioles, I38 Unspecified valve endocarditis, I44 Atrioventricular and left bundle branch block, I33 Acute and subacute endocarditis, I34 Non-rheumatic mitral valve disorders, I05 Rheumatic mitral valve diseases, I62 Other non-traumatic intracranial hemorrhages, I74 Arterial embolism and thrombosis, I46 Cardiac arrest, I27 Other forms of pulmonary heart disease, I13 Hypertensive heart and kidney disease, I73 Other peripheral vascular diseases, I35 Non-rheumatic aortic valve disorders, I70 Atherosclerosis, I48 Atrial flutter and fibrillation, I12 Hypertensive renal disease, I49 Other cardiac arrhythmias, I24 Other acute ischemic heart disease, I60 Subarachnoid hemorrhage, I51 Complications of heart disease and poorly defined heart conditions, I26 Pulmonary embolism, I71 Aortic aneurysm and dissection, I63 Cerebral infarction, I67 Other cerebrovascular diseases, I42 Cardiomyopathies, I69 Sequelae of cerebrovascular diseases, I61 Intracerebral hemorrhage, I11 Hypertensive heart disease, I25 Chronic ischemic heart disease, I10 Essential (primary) hypertension, I50 Heart failure, I64 Stroke, not specified as hemorrhagic or ischemic, and I21 Acute myocardial infarction. The graphs show the relationship between them every nine years. Only the diseases highlighted in red, corresponding to the most prevalent causes of CVD mortality, are further analysed and discussed in the manuscript. (**B**) Timeline of the seven most common causes of CVD death per 100,000 inhabitants, from 1996 to 2023. Acute myocardial infarction (I21) and hypertensive heart disease (I11) have become the most common causes of CVD death. Heart failure (I50) remains a concerning cause of death. (**C**) Number of deaths from the seven most common CVDs in Brazil per 100,000 inhabitants varies according to geographic region. All regions show an increase in mortality from acute myocardial infarction (I21), which is the most common cause of CVD death in all regions.

Among the selected conditions, acute myocardial infarction remained the leading cause of death throughout the study period. Heart failure ranked second until 2016, after which it was overtaken by hypertensive diseases, which remained the second leading cause of mortality through 2023 (Fig. 2B). This shift occurred at different times across Brazilian regions, emerging earlier in the Northeast (around 2007) and after 2014 in the remaining regions (Fig. 2C).

Regarding sex-specific mortality, acute myocardial infarction remained the leading cause of death in both men and women between 1996 and 2023, with mortality rates consistently higher in men (approximately 21.1–26.5 deaths per 100,000 inhabitants) than in women (14.6–17.6 deaths per 100,000 inhabitants). Heart failure and hypertensive diseases were the second and third leading causes of death, respectively. Heart failure mortality declined steadily in both sexes, decreasing from approximately 10 to 7.5 deaths per 100,000 inhabitants over the study period. In contrast, mortality from hypertensive diseases increased markedly, rising from about 3 to nearly 10 deaths per 100,000 inhabitants, with consistently higher rates among women than men, particularly in the later years of the series. Records with unavailable sex information remained consistently low throughout the study period (Fig. 3A).

**Fig. 3:**
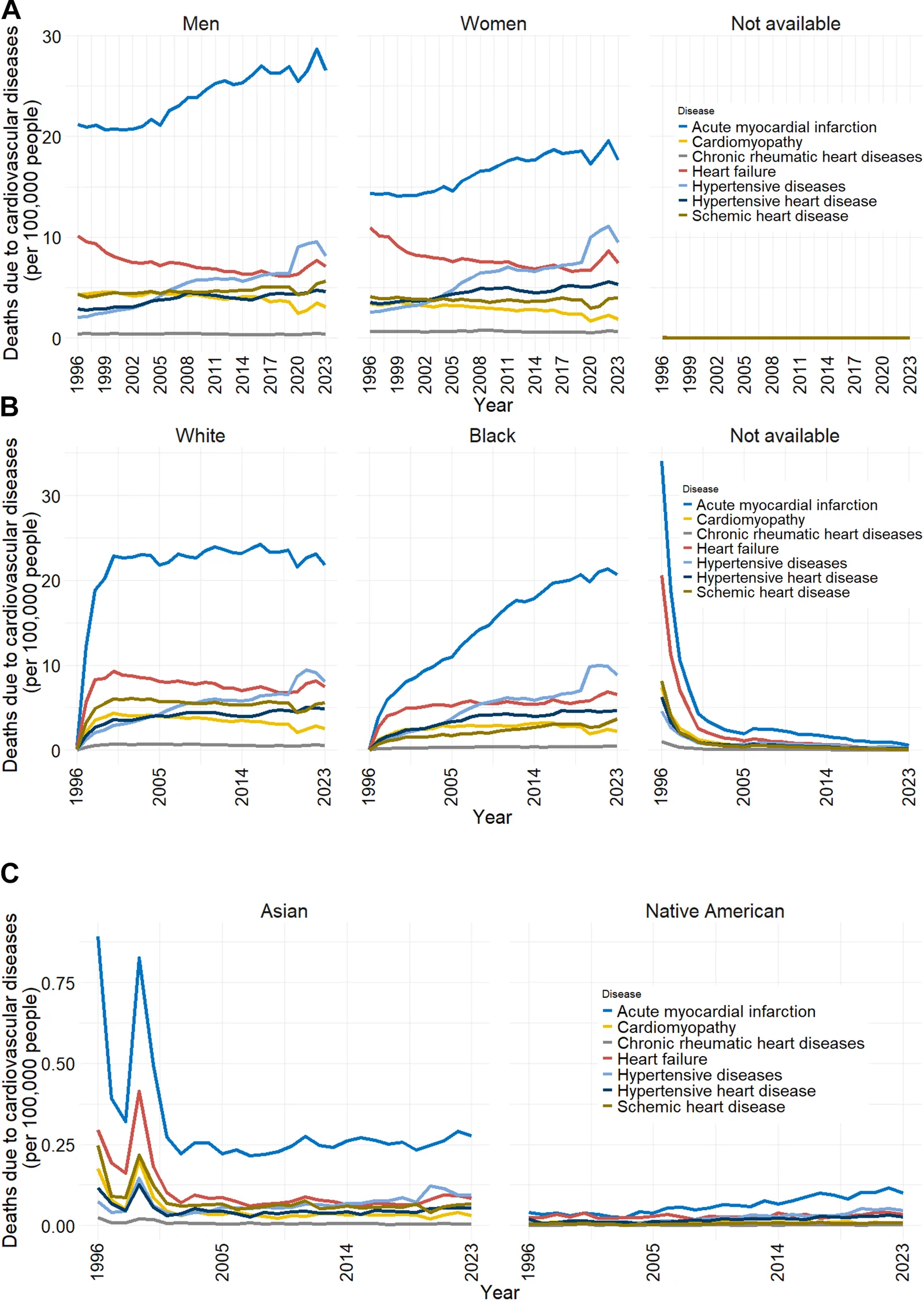
**Profile of selected cardiovascular diseases (CVD) according to sex and ethnicity in Brazil.** (**A**) Temporal trends in mortality from the seven selected CVDs according to sex. Acute myocardial infarction remained the leading cause of death in both sexes and consistently accounted for substantially more deaths in men than in women, whereas the remaining CVDs showed smaller sex-related differences. (**B**) Temporal trends in mortality according to self-reported ethnicity (White, Black, and Not available). Acute myocardial infarction was the leading cause of death in all groups, while hypertensive diseases contributed proportionally more to mortality among Black individuals. (**C**) Temporal trends in mortality among individuals of Asian descent and Indigenous populations. Mortality rates were considerably lower than those observed in the other ethnic groups, although acute myocardial infarction remained the predominant cause of death. Mortality rates are expressed as deaths per 100,000 inhabitants.

Regarding racial differences in CVD mortality, white and black populations exhibited distinct temporal patterns. Among white individuals, mortality from acute myocardial infarction increased sharply during the late 1990s and subsequently remained relatively stable at high levels, whereas among black individuals it showed a gradual and sustained increase throughout the study period. In both populations, mortality from hypertensive diseases progressively increased, whereas heart failure mortality declined only among white individuals. Consequently, hypertensive diseases overtook heart failure as the second leading cause of death earlier in the black population and later among whites. The proportion of records classified as “not available” decreased markedly over time, likely reflecting improvements in death certificate reporting (Fig. 3B).

Among Asian populations, acute myocardial infarction also emerged as the leading cause of death, although mortality estimates were more variable during the early years of the study. The ranking of the remaining diseases was less consistent, with hypertensive diseases becoming the second leading cause toward the end of the study period (Fig. 3C). Mortality data for Native American populations were highly variable throughout the series, likely owing to the small number of recorded deaths, although acute myocardial infarction became the predominant cause of death in the later years (Fig. 3C).

We also investigated the impact of CVD across age groups. CVD-related mortality remained low among individuals younger than 50 years, increased progressively from 50 to 59 years onward, and reached its highest levels among those aged 80 years or older. Despite this age-dependent pattern, overall CVD mortality declined across all age groups between 1996 and 2023, with the largest reduction observed in individuals aged 80+ years (Fig. 4A).

**Fig. 4:**
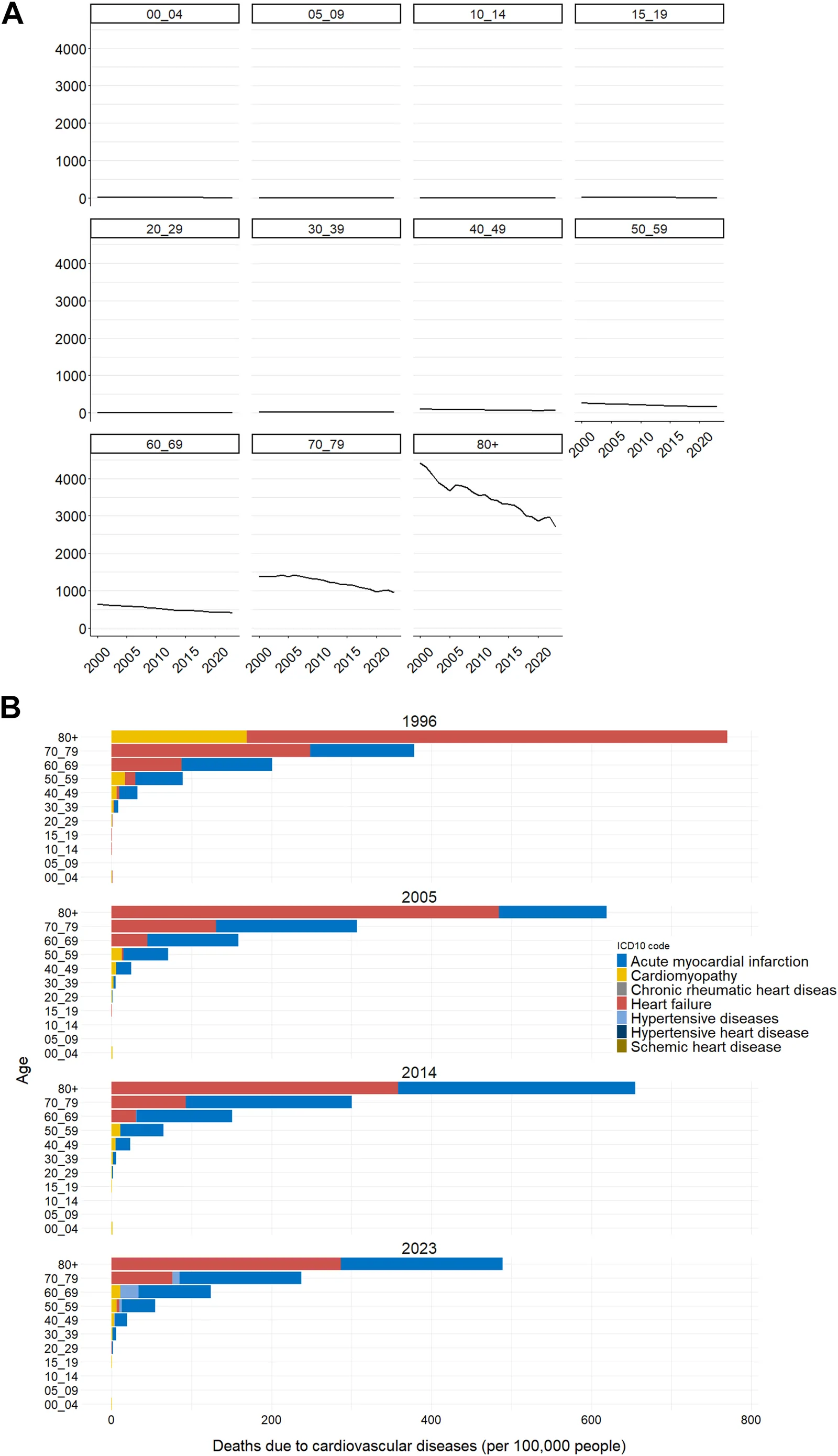
**Age-specific patterns of cardiovascular disease (CVD) mortality in Brazil.** (**A**) Temporal trends in age-specific CVD mortality rates from 1996 to 2023. Mortality increased markedly with advancing age, particularly after 50 years, while remaining relatively stable over time within each age group. The ≥80-year group exhibited the highest mortality rates throughout the study period, despite a gradual decline over time. Lines represent the mean mortality rate and shaded areas indicate the 95% confidence interval. (**B**) Distribution of the leading causes of CVD death according to age group in 1996, 2005, 2014, and 2023. Acute myocardial infarction was the leading cause of death from 30 years of age onward, whereas heart failure predominated among individuals aged ≥80 years throughout the analysed period.

When analysing causes of death across age groups in 1996, heart failure was the leading cause among individuals aged 70 years or older, whereas acute myocardial infarction was the leading cause among those aged 30–69 years. The second leading cause varied across age groups: cardiomyopathy ranked second among individuals aged 80 years or older and those younger than 60 years, whereas acute myocardial infarction ranked second among individuals aged 60–79 years (Fig. 4B).

By 2005, acute myocardial infarction had become the leading cause of death among individuals aged 40–79 years and had increased substantially among those aged 80 years or older, becoming the second leading cause of death in this age group. By 2014, it accounted for nearly half of CVD-related deaths among individuals aged 80 years or older. In 2023, it remained the leading cause of death among individuals aged 30–79 years and the second leading cause among those aged 80 years or older. Heart failure remained the leading cause of death in the oldest age group throughout the study period (Fig. 4B).

Importantly, from 1996 to 2023, both disease-specific and overall CVD mortality rates declined across age groups, particularly among older individuals. In 80+ years individuals, mortality rates decreased from nearly 800 deaths per 100,000 inhabitants in 1996 to less than 500 per 100,000 in 2023. Similarly, among individuals aged 70–79 years, mortality rates declined from nearly 400 deaths per 100,000 inhabitants in 1996 to less than 250 per 100,000 in 2023 (Fig. 4B).

### The economic burden of cardiovascular diseases in Brazil

The overall expenditure associated with CVD-related hospitalizations increased in Brazil between 1998 and 2023 (Fig. 5A), although the magnitude of this increase varied considerably across its regions (Fig. 5B). The Southeast region consistently accounted for the highest hospitalization costs, increasing from approximately BRL 1.45 billion (≈ USD 283 million) in 1998 to more than BRL 2.10 billion (≈ USD 410 million) in 2023. Similarly, expenditures in the South and Northeast regions increased from BRL 640.9 million (≈ USD 125 million) to BRL 1.09 billion (≈ USD 213 million), and from BRL 487.8 million (≈ USD 95 million) to BRL 900.9 million (≈ USD 176 million), respectively. Despite presenting the lowest absolute cost compared with the other regions, the North region showed the highest increase, with expenditures rising approximately 3-fold between 1998 and 2023 (Fig. 5B).

**Fig. 5:**
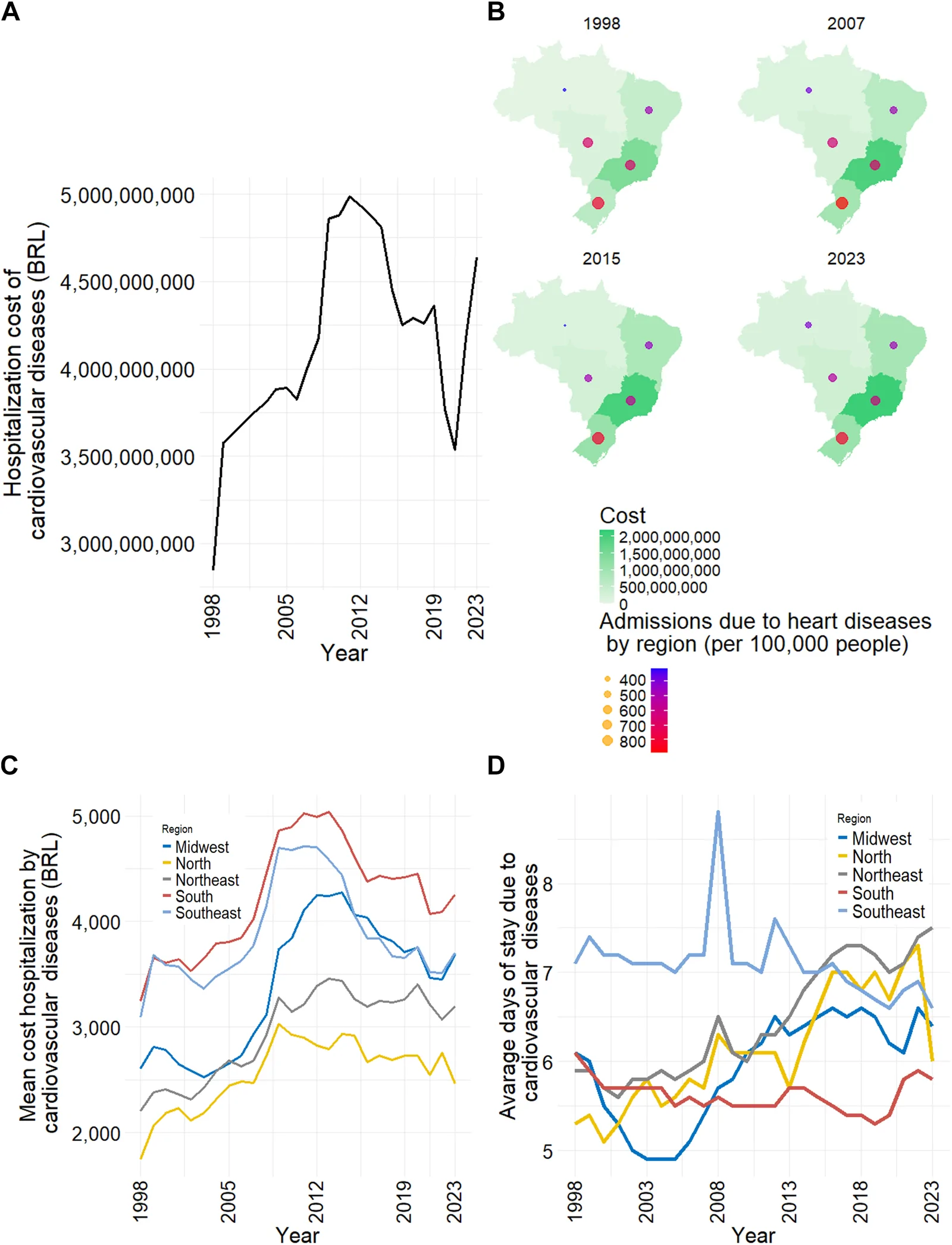
**Hospitalizations and healthcare costs associated with cardiovascular diseases (CVD) in Brazil.** (**A**) Total annual expenditure on CVD-related hospitalizations from 1998 to 2023. Values were adjusted for inflation using the Extended National Consumer Price Index (Índice Nacional de Preços ao Consumidor Amplo, IPCA) calculated by the Brazilian Institute of Geography and Statistics (IBGE). (**B**) Regional trends in total CVD-related hospitalization expenditure and hospital admissions per 100,000 inhabitants from 1998 to 2023. (**C**) Mean cost per CVD-related hospitalization according to Brazil's geographic regions between 1998 and 2023. (**D**) Average length of CVD-related hospital stay according to Brazil's geographic regions between 1998 and 2023.

CVD-related hospital admissions per 100,000 inhabitants displayed distinct regional patterns. The Midwest, Northeast and Southeast regions showed an initial reduction up to 2015, followed by a modest increase in 2023. The South region had an early increase up to 2007, followed by a slight decline, reaching rates similar to 1998. The North region showed a continuous increase throughout the period, rising from 322.3 to 417.7 admissions per 100,000 inhabitants from 1998 to 2023 (Fig. 5B). Detailed CVD-related hospitalization costs and admission rates for each Brazilian region are presented in Table S5 (supplemental Material).

All five regions showed a similar trend in the mean cost of hospitalization, with costs increasing until the early 2010s, when they reached their peak values, followed by a gradual decline through 2023. The South and Southeast regions consistently exhibited the highest mean hospitalization costs throughout the study period, whereas the North region showed the lowest values. The Midwest experienced a marked increase between 2005 and 2013, reaching levels comparable to those observed in the Southeast (Fig. 5C).

The average length of CVD-related hospital stay remained relatively stable across most Brazilian regions throughout the analysed period (Fig. 5D). The North and Northeast regions were exceptions, showing a gradual increase over time. In the North, the average stay rose from approximately 5.4 days in 1998 to 6.0 days in 2023, peaking at 7.3 days in 2022, whereas in the Northeast it increased steadily from 5.8 to 7.5 days over the same period. The South region remained comparatively stable, fluctuating between 5.3 and 6.0 days and exhibiting the shortest hospital stays by 2023. The Midwest showed an initial decline followed by a gradual increase after 2010, whereas the Southeast consistently presented the longest hospital stays until around 2016, when it was surpassed by the Northeast, which thereafter exhibited the longest average length of stay among all Brazilian regions.

### Heart transplant panorama in Brazil

We analysed the impact of heart transplantation on Brazil's public health system (SUS), as it represents a last-line therapy for patients with severe, treatment-refractory heart disease (Fig. 6). It is important to note that the unusually low number of heart transplants recorded in 2007 should be interpreted with caution, as this period coincided with the implementation of a new DATASUS procedure coding system and the unification of hospital and outpatient information databases, which likely affected data reporting and comparability during the transition. Since then, the number of heart transplants progressively increased, reaching a peak of 0.162 transplants per 100,000 inhabitants in 2019 and remaining at similarly high levels in 2023 (0.136 per 100,000 inhabitants) (Fig. 6A). The number of transplantation-related intercurrences also increased over time, rising from fewer than 0.05 cases per 100,000 inhabitants in 2012 to approximately 0.4 in 2023, when it reached its highest level (Fig. 6B).

**Fig. 6:**
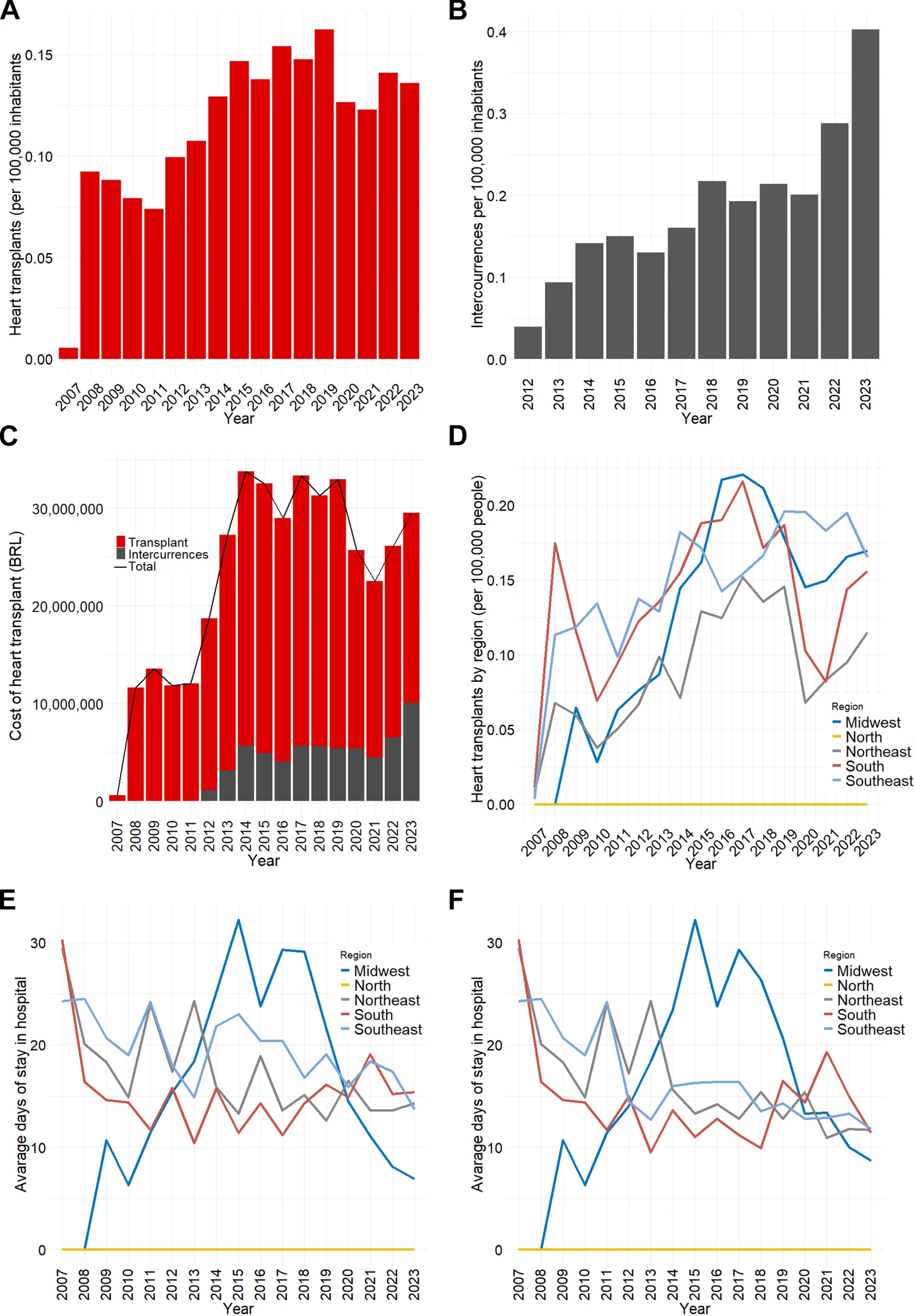
**Heart transplantation in Brazil from 2007 to 2023.** (**A**) Annual number of heart transplants per 100,000 inhabitants in Brazil. (**B**) Annual number of heart transplantation-related intercurrences per 100,000 inhabitants from 2012 to 2023. (**C**) Total annual expenditure on heart transplantation in Brazil (BRL). Values were adjusted for inflation using the Extended National Consumer Price Index (“Índice Nacional de Preços ao Consumidor Amplo”, IPCA) calculated by the Brazilian Institute of Geography and Statistics (IBGE). (**D**) Heart transplantation rate (per 100,000 inhabitants) according to Brazil's geographic regions from 2007 to 2023. (**E**) Average length of hospital stay following heart transplantation according to geographic region. (**F**) Average length of hospital stay associated with heart transplantation-related intercurrences according to geographic region.

The total expenditure on heart transplantation peaked in 2014, reaching BRL 33,807,575.20 (≈ USD 6.6 million). Costs remained relatively high until 2020, when a temporary decline was observed, before increasing again through 2023 (Fig. 6C).

Between 2007 and 2023, heart transplantation rates generally increased across the Brazilian regions, although substantial year-to-year fluctuations were observed. The Midwest, South, and Northeast regions reached their highest rates between 2016 and 2018, followed by a decline during 2020–2021. In contrast, the Southeast peaked around 2018–2019 and maintained relatively high transplantation rates thereafter. Notably, the North region recorded no heart transplants throughout the study period (Fig. 6D).

The average length of hospitalization for heart transplantation (Fig. 6E) and transplantation-related intercurrences (Fig. 6F) showed very similar regional patterns throughout the analysed period. The South, Southeast, and Northeast regions exhibited an overall decline in hospitalization duration, although with substantial year-to-year fluctuations. In the South and Northeast, the average length of stay decreased from approximately 30 days in 2007 to around 15 days by 2023, whereas the Southeast declined from approximately 25 to 15 days over the same period. In contrast, the Midwest displayed a distinct pattern, with hospitalization duration increasing sharply between 2010 and 2015, peaking at more than 30 days, before declining to fewer than 10 days by 2023. As expected, the North region consistently recorded zero hospitalization days due to the absence of heart transplant procedures during the study period (Fig. 6E and F).

## Discussion

The epidemiological profile of Brazil has changed dramatically over the past three decades. In the early 1990s, communicable diseases (i.e., diarrhoeal diseases) were the leading causes of mortality,^8^ largely reflecting inadequate sanitation and the early stages of implementation of the Unified Health System (SUS).^19^^,^^20^ Since then, Brazil has undergone a marked epidemiological transition, with CVD progressively becoming the leading cause of death.^8^ In this context, our study provides a comprehensive longitudinal assessment of the burden of CVD in Brazil over nearly three decades (1996–2023). While GBD studies provided valuable modelled estimates of CVD burden in Brazil,^8^^,^^9^ our study complements these efforts by directly analysing official nationwide administrative databases from the Brazilian Unified Health System (DATASUS). By integrating nationwide data on mortality, hospitalizations, healthcare expenditures, length of hospital stay, heart transplantation, and demographic and regional disparities over a 27-year period, our study provides a health-system perspective that extends previous GBD analyses and offers a detailed characterization of the burden of CVD in Brazil. Furthermore, our study expands a previous DATASUS-based report focusing primarily on CVD-related emergency hospitalizations between 2013 and 2023^21^ by providing a comprehensive assessment of the ecological and economic burden of CVD and its implications for public health planning and resource allocation. This long-term analysis also allowed the identification of important temporal disruptions in CVD indicators, particularly during the COVID-19 pandemic. A marked reduction in several indicators was observed in 2020, likely reflecting disruptions in health information systems and changes in healthcare-seeking behaviour during the COVID-19 pandemic. The subsequent rebound may also reflect the indirect cardiovascular consequences of delayed diagnosis and treatment during this period.^22^, ^23^, ^24^ Although CVD-related healthcare expenditures increased again after 2020, they did not return to the peak levels observed before the pandemic, suggesting a persistent impact on healthcare utilisation and resource allocation.

Between 1996 and 2023, CVD deaths in Brazil (Human Development Index [HDI] = 0.805) increased, reaching 383,474 deaths in 2023, approximately 180.0 per 100,000 inhabitants. By comparison, the United States (HDI = 0.938) reported 129.5 deaths per 100,000 inhabitants in 2022, France (HDI = 0.920) 62.6, whereas Uzbekistan (HDI = 0.740) recorded the highest rate worldwide in 2023, with 594.1 deaths per 100,000 inhabitants. Although multiple factors contribute to these differences, these comparisons are consistent with an inverse association between socioeconomic development and CVD mortality, which has been reported globally^25^^,^^26^ and within Brazilian cities.^9^^,^^27^

Regional disparities in the burden of CVD were among the most striking findings of this study. Brazil encompasses a vast territorial area of approximately 8.51 million km^2^ and an estimated population of 213.4 million inhabitants, distributed across five official geographic regions (North, Northeast, Midwest, Southeast, and South). These regions exhibit marked differences in population size, demographic characteristics, socioeconomic development, and healthcare infrastructure,^9^^,^^28^ all of which directly influence the epidemiological patterns analysed in this study. In general, the South and Southeast regions have older populations and are associated with better access to healthcare services, infrastructure, and economic opportunities than the North and Northeast. To illustrate the socioeconomic variation across Brazilian regions, the Gross Domestic Product (GDP) of the Southeast region was approximately BRL 5,889,593 million (≈ USD 1,150,828 million) while the North region generated BRL 636,552 million (≈ USD 124,382 million) in 2023, highlighting the marked economic disparities across the country.^29^

The Southeast and South regions consistently recorded the highest numbers of CVD-related deaths, probably reflecting their larger and older populations.^30^ However, when mortality rates were considered, the Northeast region exhibited the highest CVD mortality rate in 2023. Nonetheless, lower mortality rates observed in other regions should be interpreted cautiously, as they may partly reflect underdiagnosis and underreporting. Evidence from municipalities in the Amazon area, in the Nort region, has demonstrated substantial discrepancies between local notification systems and the national DATASUS database, exposing important limitations in disease registration and surveillance.^31^ This issue is particularly relevant for the North region, which simultaneously showed the largest increase in CVD-related hospitalizations yet recorded no heart transplants throughout the study period. Together with its lower mortality rates, these findings highlight persistent inequalities in access to highly specialised cardiovascular care and suggest that both healthcare availability and information system limitations require greater attention in this region. They may also reflect important differences in healthcare system capacity, including the unequal distribution of specialised cardiovascular centres, diagnostic services, and heart transplantation programmes across the country.^9^

This issue can be understood in light of the Brazil's historical context, which concentrated political and economic centres in the Southeast region while sidelining more inland areas, such as the North. This historical legacy appears to persist today, as reflected by the ongoing challenges in achieving equitable healthcare across Brazilian regions.^32^ Consistent with previous reports,^9^ our findings further support the need for region-specific healthcare planning and resource allocation. Expanding access to specialised cardiovascular services and strengthening health information systems in underserved areas may contribute to reducing regional disparities in the burden and management of CVD.

It is worth noting that urban and highly industrialised centres, particularly state capitals, tend to concentrate a greater number of risk factors for CVD. Prominent among these factors are high levels of air pollution and exposure to nitrogen dioxide (NO_2_), which have been associated with increased cardiovascular risk. Furthermore, noise pollution and other characteristics of the urban environment may contribute to chronic stress and increase cardiovascular risk.^33^ Although these factors were not directly evaluated in the present study, they may partly contribute to the regional differences observed, particularly in highly urbanised regions.

The Brazilian Institute of Geography and Statistics (IBGE) classifies ethnicity into five groups (Black, Brown, Asian, Indigenous, and White) based on self-declared skin colour. In epidemiological studies, Black and Brown populations are commonly combined to represent the Black population, an approach adopted here. Within this framework, hypertensive disease mortality increased disproportionately among Black individuals. Although genetic susceptibility has been proposed, the reasons underlying these disparities are likely multifactorial and cannot be determined from the present study. It is plausible that structural racism, unequal access to healthcare, delayed diagnosis, differences in treatment, and socioeconomic inequalities contribute to the observed differences in mortality.^34^, ^35^, ^36^, ^37^ However, these hypotheses could not be directly evaluated using the aggregated administrative data analysed in this study.

Similarly, the markedly lower number of CVD-related cases observed among Indigenous populations is likely influenced by underreporting, a limitation previously described in other epidemiological studies in Brazil.^38^^,^^39^ Geographic isolation, barriers to healthcare access, and limited health infrastructure may contribute to incomplete case registration, suggesting that the true burden of CVD in these communities is substantially underestimated.

Sex and age emerged as major determinants of CVD mortality in the present study, corroborating previous evidence that both are major determinants of cardiovascular risk and mortality.^40^^,^^41^ Acute myocardial infarction consistently accounted for substantially more deaths in men than in women, whereas sex differences for other CVDs were less pronounced. This male predominance may reflect both greater exposure to behavioural risk factors and an intrinsically faster biological ageing process, which has recently been shown to explain a substantial proportion of the excess cardiovascular risk observed in men, independent of traditional risk factors and sex hormones.^42^ Age had an even greater influence on CVD mortality, with death rates increasing exponentially after 50 years of age. Heart failure predominated among the oldest individuals, whereas acute myocardial infarction became the leading cause of death from 30 years of age onward, highlighting distinct age-related patterns in the burden of CVD.

### Study limitations and strengths

A major strength of this study is the systematic analysis of nationwide CVD data spanning nearly three decades. The use of standardized national administrative databases enabled comprehensive comparisons across geographic regions, age groups, sex, and race/ethnicity using a consistent methodology. Previous validation studies have shown that the Brazilian Mortality Information System (SIM) and Hospital Information System (SIH/SUS) have progressively improved in coverage, completeness, and reliability, supporting the overall validity of the data used in this study. Nevertheless, these strengths should be interpreted in light of several important limitations. First, because analyses were based on aggregated administrative data, individual-level information was unavailable, precluding adjustment for potential confounding factors such as socioeconomic characteristics, cardiovascular risk factors, and pre-existing medical conditions. Second, the use of administrative databases introduces the possibility of information bias resulting from underreporting, incomplete records, and diagnostic or coding misclassification, although these limitations have decreased over time as the national information systems have improved. Third, morbidity data were restricted to hospitalizations financed by the Brazilian Unified Health System (SUS), which, although covering most of the Brazilian population, does not capture all admissions occurring in the private healthcare sector, potentially leading to underestimation of hospitalization rates and healthcare expenditures. In addition, the absence of individual patient records prevented patient follow-up, differentiation between incident admissions and readmissions, calculation of disability-adjusted life years (DALYs), and evaluation of disease progression. Finally, simultaneous stratification by age and sex was not possible using the aggregated data available, limiting more detailed subgroup analyses.

### Conclusion

CVD remains a leading cause of death in Brazil and worldwide, imposing heavy epidemiological and economic burdens. Mortality rates stay high across Brazil, with disparities by time, region, ethnicity, sex, and age. Acute myocardial infarction dominates, while hypertension and heart failure also contribute significantly. The study highlights rising hospitalization and transplant costs, regional inequalities in access and reporting, and the urgent need for stronger surveillance, equitable care, and preventive strategies targeting vulnerable groups and modifiable risks.

## Contributors

AGO and TVB conceptualized the idea of the analysis and research, with input from all authors. AGO and TVB were responsible for funding acquisition, determining the methodology for this study and project administration. MCBS and JSFG performed data curation and investigation of all the data in this study, with input from ALMO, AGO and TVB. MCBS, JSFG, TVB, and AGO were responsible for the formal analysis with contributions from all authors. ALMO conducted data curation for correction of costs based on the national extended consumer price index throughout the years. MCBS, JSFG and TVB have directly accessed and verified the underlying data. MCBS, JSFG were responsible for writing the original draft, with significant contributions from all authors. MCBS, JSFG TVB, and AGO were responsible for writing-reviewing and editing the final version of this draft, with significant contributions from all authors. All authors reviewed and approved the final manuscript and are responsible for the decision to submit for publication.

## Data sharing statement

The data used for this study were retrieved from public online repositories maintained by the Brazilian Government. The system allows anyone who wishes to access the data (https://datasus.saude.gov.br/informacoes-de-saude-tabnet/). Data can be retrieved from different sets as follows: Morbidity (https://datasus.saude.gov.br/acesso-a-informacao/morbidade-hospitalar-do-sus-sih-sus/), Mortality (http://tabnet.datasus.gov.br/cgi/deftohtm.exe?sim/cnv/obt10uf.def), and Transplants (http://tabnet.datasus.gov.br/cgi/deftohtm.exe?sih/cnv/qruf.def).

## Use of generative AI or AI-assisted technologies

The authors used ChatGPT (OpenAI) to assist with language editing and improve the clarity and readability of the text. The authors reviewed and edited all AI-assisted content and take full responsibility for the final content of the manuscript.

## Editor note

The Lancet Group takes a neutral position with respect to territorial claims in published maps and institutional affiliations.

## Declaration of interests

Authors declare no competing interests.
