# Supplementary material for: Burden of cardiovascular disease in Brazil, 1996–2023: a retrospective descriptive study of the epidemiology and impact on public healthcare
Source: Lancet Reg Health Am. 2026 Oct 14;65:101674. doi: 10.1016/j.lana.2026.101674 (PMC13683861; doi:10.1016/j.lana.2026.101674)
Supplement: Supplementary file 1 [file mmc1.pdf]

Supplementary Table S1: Heart diseases defined by the International Classification of Diseases (ICD10)

Code	ICD-10 Category
I00	Rheumatic fever without mention of heart involvement
I01	Rheumatic fever with heart involvement
I02	Rheumatic chorea
I05	Rheumatic mitral valve diseases
I06	Rheumatic aortic valve diseases
I07	Rheumatic tricuspid valve diseases
I08	Multiple valve diseases
I09	Other rheumatic heart diseases
I10	Essential (primary) hypertension
I11	Hypertensive heart disease
I13	Hypertensive heart and renal disease
I15	Secondary hypertension
I20	Angina pectoris
I21	Acute myocardial infarction
I22	Subsequent myocardial infarction
I23	Certain current complications following acute myocardial infarction
I24	Certain current complications following acute myocardial infarction
I25	Chronic ischaemic heart disease
I26	Pulmonary embolism
I27	Other pulmonary heart diseases
I28	Other diseases of pulmonary vessels
I30	Acute pericarditis
I31	Other diseases of pericardium
I33	Acute and subacute endocarditis
I34	Nonrheumatic mitral valve disorders

I35	Nonrheumatic aortic valve disorders
I36	Nonrheumatic tricuspid valve disorders
I37	Pulmonary valve disorders
I38	Endocarditis, valve unspecified
I40	Acute myocarditis
I42	Cardiomyopathy
I44	Atrioventricular and left bundle-branch block
I45	Other conduction disorders
I46	Cardiac arrest
I47	Paroxysmal tachycardia
I48	Atrial fibrillation and flutter
I49	Other cardiac arrhythmias
I50	Heart failure
I51	Complications and ill-defined descriptions of heart disease
I60	Subarachnoid haemorrhage
I61	Intracerebral haemorrhage
I62	Other nontraumatic intracranial haemorrhage
I63	Cerebral infarction
I64	Stroke, not specified as haemorrhage or infarction
I66	Occlusion and stenosis of cerebral arteries, not resulting in cerebral infarction
I67	Other cerebrovascular diseases
I69	Sequelae of cerebrovascular disease
I70	Atherosclerosis
I71	Aortic aneurysm and dissection
I72	Other aneurysm and dissection
I73	Other peripheral vascular diseases
I74	Arterial embolism and thrombosis

I77	Other disorders of arteries and arterioles
I78	Diseases of capillaries

Supplementary Table S2: Description of variables analysed

Variable	Description
Sex	Sex Sex of the patient (male, female)
Race/ethnicity	Race/ethnicity of the patient (White, Black, Brown, Asian, Indigenous, or unspecified)
ICD10 chapter	Cause of hospitalization, according to the International Classification of Diseases (ICD),retrieved by chapters.
ICD10 list	Cause of hospitalization, according to the International Classification of Diseases (ICD),retrieved by list.
ICD10 Category	Cause of hospitalization, according to the International Classification of Diseases (ICD),retrieved by category .
Age group	Under 1 year, 1–4 years, 5–9 years, 10–14 years, 15–19 years, 20–29 years, 30–39 years, 40–49 years, 50–59 years, 60–69 years, 70–79 years, 80 years and older.

Supplementary Table S3: Description of parameters analysed

Parameters	Description
Hospitalization admission	Number of HAA (Hospital Admission Authorization) approved during the period,excluding extensions (long-stay cases). This is an approximate value of hospitalizations, as transfers and readmissions are included in this count
Total hospitalization cost	The value referring to the approved HAA (Hospital Admission Authorization) during the period. This value may not necessarily correspond to the amount transferred to the healthcare facility, as it depends on the situation of the units. They may receive budgetary resources or there may be retentions and payments of incentives, which are not presented here. Therefore, this value should be considered as the approved production value
Mean cost of hospitalization admission	Total hospitalization cost divided by the number of hospitalizations
Average days of stay in hospital	Average length of stay for hospitalizations related to approved HAA (HospitalAdmission Authorization), recorded as admissions, during the period.
Deaths	Number of hospitalizations that resulted in discharge due to death among the approvedHAA (Hospital Admission Authorization) during the period.
Mortality rate	The ratio between the number of deaths and the number of approved HAA (HospitalAdmission Authorization) recorded as hospitalizations during the period, multiplied by100.

Supplementary Table S4: Hospital Morbidity in the Unified Health System of Brazil

Chapter	Code	Description	ICD10
IX	066-072	Diseases of the circulatory system	I00-I99

Supplementary Table S5: Relative admissions and hospitalisation cost by regions

Year	Region	Admissions (per 100,000)	Cost (in BRL)
1998	Midwest	672.7167	192,959,555
1998	North	322.2924	66,846,381
1998	Northeast	482.8149	487,769,710
1998	South	817.8246	640,926,201
1998	Southeast	681.7196	1,453,770,982
2007	Midwest	645.6512	250,704,331
2007	North	395.4090	143,000,080
2007	Northeast	483.5087	669,003,818
2007	South	893.0429	960,454,497
2007	Southeast	675.8243	1,986,099,584
2015	Midwest	493.9312	309,871,057
2015	North	316.3253	161,921,327
2015	Northeast	456.1804	843,431,002
2015	South	827.7951	1,117,167,726
2015	Southeast	579.4985	2,018,729,278
2023	Midwest	541.0276	341,468,367
2023	North	417.6548	199,353,135
2023	Northeast	483.4854	900,931,365
2023	South	829.4803	1,086,713,935
2023	Southeast	627.2055	2,108,607,004
