# Supplementary material for: Burden of cardiovascular disease in Brazil, 1996–2023: a retrospective descriptive study of the epidemiology and impact on public healthcare
Source: Lancet Reg Health Am. 2026 Oct 14;65:101674. doi: 10.1016/j.lana.2026.101674 (PMC13683861; doi:10.1016/j.lana.2026.101674)
Supplement: Supplementary file 2 [file mmc2.pdf]

# Table of content

**Supplementary Table S1: Description of variables analysed** 2
**Supplementary Table S2: Description of parameters analysed** 2
**Supplementary Table S3: Hospital Morbidity in the Unified Health System of Brazil** 3
**Supplementary Table S4: Sociodemographic characteristics of the Brazilian regions** 3
**Supplementary Table S5: Relative admissions and hospitalisation cost by regions** 5

**Supplementary Table S1:** Description of variables analysed

| Variable | Description |
|---|---|
| Sex | Sex of the patient (male, female) |
| Race/ethnicity | Race/ethnicity of the patient (White, Black, Brown, Asian, Indigenous, or unspecified) |
| ICD10 chapter | Cause of hospitalization, according to the International Classification of Diseases (ICD),retrieved by chapters. |
| ICD10 list | Cause of hospitalization, according to the International Classification of Diseases (ICD),retrieved by list. |
| ICD10 Category | Cause of hospitalization, according to the International Classification of Diseases (ICD),retrieved by category . |
| Age group | Under 1 year, 1–4 years, 5–9 years, 10–14 years, 15–19 years, 20–29 years, 30–39 years, 40–49 years, 50–59 years, 60–69 years, 70–79 years, 80 years and older. |

**Supplementary Table S2:** Description of parameters analysed

| Parameters | Description |
|---|---|
| Hospitalization admission | Number of HAA (Hospital Admission Authorization) approved during the period,excluding extensions (long-stay cases). This is an approximate value of hospitalizations, as transfers and readmissions are included in this count |
| Total hospitalization cost | The value referring to the approved HAA (Hospital Admission Authorization) during the period. This value may not necessarily correspond to the amount transferred to the healthcare facility, as it depends on the situation of the units. They may receive budgetary resources or there may be retentions and payments of incentives, which are not presented here. Therefore, this value should be considered as the |

| | |
|---|---|
| | approved production value |
| Mean cost of hospitalization admission | Total hospitalization cost divided by the number of hospitalizations |
| Average days of stay in hospital | Average length of stay for hospitalizations related to approved HAA (HospitalAdmission Authorization), recorded as admissions, during the period. |
| Deaths | Number of hospitalizations that resulted in discharge due to death among the approvedHAA (Hospital Admission Authorization) during the period. |
| Mortality rate | The ratio between the number of deaths and the number of approved HAA (HospitalAdmission Authorization) recorded as hospitalizations during the period, multiplied by100. |

**Supplementary Table S3:** Hospital Morbidity in the Unified Health System of Brazil

| Chapter | Code | Description | ICD10 |
|---|---|---|---|
| IX | 066-072 | Diseases of the circulatory system | I00-I99 |

**Supplementary Table S4:** Sociodemographic characteristics of the Brazilian regions

| State | Region | Population | GDP (1000 BRL) | GDP *per capita* (BRL) | GDP *per capita* (USD) | MHDI |
|---|---|---|---|---|---|---|
| Acre | North | 830,018 | 23,676,136.06 | 28,524.85 | 5,573.76 | 0.732 |
| Amapá | North | 733,759 | 23,614,291.47 | 32,182.63 | 6,288.49 | 0.743 |
| Amazonas | North | 3,941,613 | 145,140,465.10 | 36,822.61 | 7,195.15 | 0.757 |
| Pará | North | 8,120,131 | 236,141,873.80 | 29,081.04 | 5,682.44 | 0.734 |

| | | | | | | |
|---|---|---|---|---|---|---|
| Rondônia | North | 1,581,196 | 66,795,453.51 | 42,243.63 | 8,254.42 | 0.762 |
| Roraima | North | 636,707 | 21,095,341.82 | 33,131.95 | 6,473.99 | 0.763 |
| Tocantins | North | 1,511,460 | 58,208,795.20 | 38,511.63 | 7,525.18 | 0.777 |
| Alagoas | Northeast | 3,127,683 | 76,065,805.84 | 24,320.18 | 4,752.17 | 0.722 |
| Bahia | Northeast | 14,141,626 | 402,646,605.60 | 28,472.44 | 5,563.52 | 0.734 |
| Ceará | Northeast | 8,794,957 | 213,600,673.80 | 24,286.72 | 4,745.63 | 0.759 |
| Maranhão | Northeast | 6,776,699 | 139,789,146.00 | 20,627.91 | 4,030.70 | 0.730 |
| Paraíba | Northeast | 3,974,687 | 86,094,173.05 | 21,660.62 | 4,232.49 | 0.737 |
| Pernambuco | Northeast | 9,058,931 | 245,828,016.70 | 27,136.54 | 5,302.49 | 0.745 |
| Piauí | Northeast | 3,271,199 | 72,834,503.56 | 22,265.38 | 4,350.66 | 0.743 |
| Rio Grande do Norte | Northeast | 3,302,729 | 93,819,291.46 | 28,406.6 | 5,550.66 | 0.744 |
| Sergipe | Northeast | 2,210,004 | 57,372,241.37 | 25,960.24 | 5,072.64 | 0.738 |
| Distrito Federal | Midwest | 2,817,381 | 328,789,563.00 | 116,700.4 | 22,803.29 | 0.867 |
| Goiás | Midwest | 7,056,495 | 318,586,149.30 | 45,147.93 | 8,821.92 | 0.797 |
| Mato Grosso | Midwest | 3,658,649 | 255,527,297.30 | 69,841.98 | 13,647.14 | 0.794 |
| Mato Grosso do Sul | Midwest | 2,757,013 | 166,407,328.50 | 60,357.83 | 11,793.94 | 0.781 |
| Espírito Santo | Southeast | 3,833,712 | 182,548,589.70 | 47,616.67 | 9,304.31 | 0.782 |
| Minas Gerais | Southeast | 20,539,989 | 906,730,670.40 | 44,144.65 | 8,625.88 | 0.793 |
| Rio de Janeiro | Southeast | 16,055,174 | 1,153,512,155.00 | 71,846.76 | 14,038.88 | 0.804 |
| São Paulo | Southeast | 44,411,238 | 3,130,333,203.00 | 70,485.16 | 13,772.82 | 0.828 |
| Paraná | South | 11,444,380 | 614,610,848.30 | 53,704.16 | 10,493.81 | 0.800 |
| Rio Grande do Sul | South | 10,882,965 | 593,633,656.20 | 54,547.05 | 10,658.51 | 0.796 |
| Santa Catarina | South | 7,610,361 | 466,274,103.50 | 61,268.33 | 11,971.85 | 0.811 |

Data of Population and GDP from IBGE; MHDI data from PNUD

**Supplementary Table S5:** Relative admissions and hospitalisation cost by regions

| Year | Region | Admissions (per 100,000) | Cost (in BRL) | Cost (in USD) |
|---|---|---|---|---|
| 1998 | Midwest | 672.7167 | 192,959,555 | 37,704,350 |
| 1998 | North | 322.2924 | 66,846,381 | 13,061,801 |
| 1998 | Northeast | 482.8149 | 487,769,710 | 95,310,336 |
| 1998 | South | 817.8246 | 640,926,201 | 640,926,201 |
| 1998 | Southeast | 681.7196 | 1,453,770,982 | 3,279,683,463 |
| 2007 | Midwest | 645.6512 | 250,704,331 | 48,987,695 |
| 2007 | North | 395.4090 | 143,000,080 | 27,942,255 |
| 2007 | Northeast | 483.5087 | 669,003,818 | 130,723,531 |
| 2007 | South | 893.0429 | 960,454,497 | 187,673,075 |
| 2007 | Southeast | 675.8243 | 1,986,099,584 | 388,084,409 |
| 2015 | Midwest | 493.9312 | 309,871,057 | 60,548,890 |
| 2015 | North | 316.3253 | 161,921,327 | 31,639,472 |
| 2015 | Northeast | 456.1804 | 843,431,002 | 164,806,651 |
| 2015 | South | 827.7951 | 1,117,167,726 | 218,294,883 |
| 2015 | Southeast | 579.4985 | 2,018,729,278 | 394,460,261 |
| 2023 | Midwest | 541.0276 | 341,468,367 | 66,723,013 |
| 2023 | North | 417.6548 | 199,353,135 | 38,953,657 |
| 2023 | Northeast | 483.4854 | 900,931,365 | 176,042,238 |
| 2023 | South | 829.4803 | 1,086,713,935 | 212,344,204 |
| 2023 | Southeast | 627.2055 | 2,108,607,004 | 412,022,393 |
